# Embedding digital chronotherapy into bioelectronic medicines

**DOI:** 10.1016/j.isci.2022.104028

**Published:** 2022-03-04

**Authors:** John E. Fleming, Vaclav Kremen, Ro'ee Gilron, Nicholas M. Gregg, Mayela Zamora, Derk-Jan Dijk, Philip A. Starr, Gregory A. Worrell, Simon Little, Timothy J. Denison

**Affiliations:** 1 Medical Research Council Brain Network Dynamics Unit, Nuffield Department of Clinical Neurosciences, University of Oxford, Mansfield Road, Oxford OX1 3TH, UK; 2 Bioelectronics Neurophysiology and Engineering Laboratory, Department of Neurology, Mayo Clinic, Rochester, MN 55905, USA; 3 Cognitive Systems and Neurosciences, Czech Institute of Informatics, Robotics and Cybernetics, Czech Technical University in Prague, Prague, Czechia; 4 Department of Physiology and Biomedical Engineering, Mayo Clinic, Rochester, MN 55905, USA; 5 Department of Neurological Surgery, University of California San Francisco, San Francisco, CA 94143, USA; 6 Institute of Biomedical Engineering, Department of Engineering Science, University of Oxford, Oxford, UK; 7 Surry Sleep Research Centre, University of Surrey, University of Surrey, Guildford, UK; 8 UK Dementia Research Institute Care Research and Technology Centre, Imperial College London and the University of Surrey, Guildford, UK; 9 Department of Neurology, University of California San Francisco, San Francisco, CA 94143, USA

**Keywords:** Biological sciences, Neuroscience, Biotechnology, Bioelectronics

## Abstract

Biological rhythms pervade physiology and pathophysiology across multiple timescales. Because of the limited sensing and algorithm capabilities of neuromodulation device technology to-date, insight into the influence of these rhythms on the efficacy of bioelectronic medicine has been infeasible. As the development of new devices begins to mitigate previous technology limitations, we propose that future devices should integrate chronobiological considerations in their control structures to maximize the benefits of neuromodulation therapy. We motivate this proposition with preliminary longitudinal data recorded from patients with Parkinson's disease and epilepsy during deep brain stimulation therapy, where periodic symptom biomarkers are synchronized to sub-daily, daily, and longer timescale rhythms. We suggest a physiological control structure for future bioelectronic devices that incorporates time-based adaptation of stimulation control, locked to patient-specific biological rhythms, as an adjunct to classical control methods and illustrate the concept with initial results from three of our recent case studies using chronotherapy-enabled prototypes.

## Graphical abstract

**Figure:**
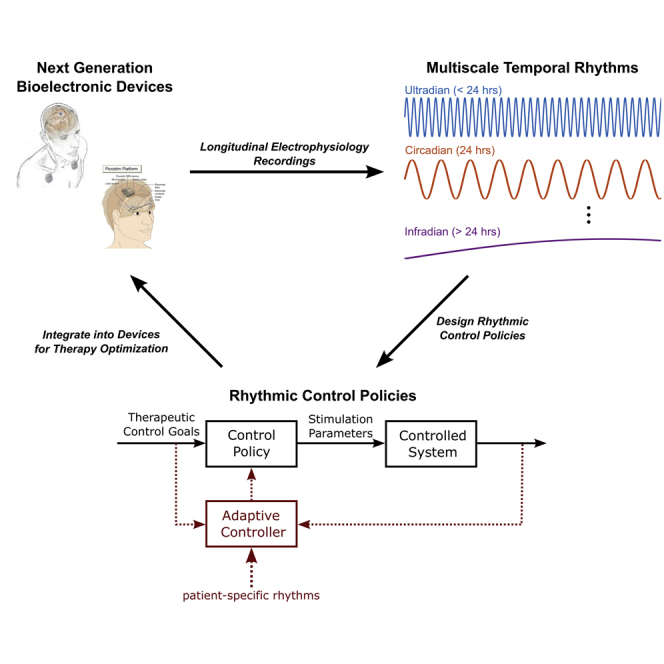

## Introduction

Throughout physiology, homeostasis is observed at multiple spatial and temporal scales. Physiological processes such as body temperature, blood pressure, and blood hormone concentrations are carefully regulated during healthy conditions, whereas dysregulation can lead to the initiation, progression, and expression of diseases (Leng et al., 2019; Smolensky and Peppas, 2007; Videnovic et al., 2014). From a control system perspective, physiological homeostasis is maintained by a combination of feedforward, feedback, and adaptive control strategies (Crago et al., 1996; Houk, 1988; Wright et al., 2016), Figure 1. In a physiological control system, a feedback controller generates a forcing function that converges the system performance to a desired setpoint by continuously comparing the control variables, such as blood pressure or glucose level, to a target setpoint. Feedback thus provides continuous negative feedback to counteract disturbances which would deviate the control variable from its target setpoint (Crago et al., 1996; Houk, 1988; Wright et al., 2016). The target setpoint is dictated by a feedforward controller which does not continuously monitor the system output. Rather, the feedforward controller operates in an open-loop where its outputs are sent as command signals to the feedback controller or directly to the controlled system as generating functions. Thus, inputs to the feedforward controller specify the overall control objectives and target set points for the control process, such as blood pressure regulation (Houk, 1988; Tan and Taylor, 2011). In addition, the feedforward controller may provide anticipatory control adjustments in response to monitored system disturbances, such as increases in blood flow during standing or exercise (Kitaoka et al., 2011; Matsukawa et al., 2012), or insulin control before eating a meal (Power and Schulkin, 2008). Feedforward and feedback control strategies alone are sufficient for the regulation of time-invariant systems however may provide suboptimal performance for time-varying systems. Inclusion of adaptive controllers are necessary for the regulation of time-varying physiological systems. The role of the adaptive controller is to modify elements of the feedforward and feedback controllers based on slow or intermittent feedback information to promote beneficial alterations in the controlled system (Crago et al., 1996; Houk, 1988; Wright et al., 2016).

**Figure 1:**
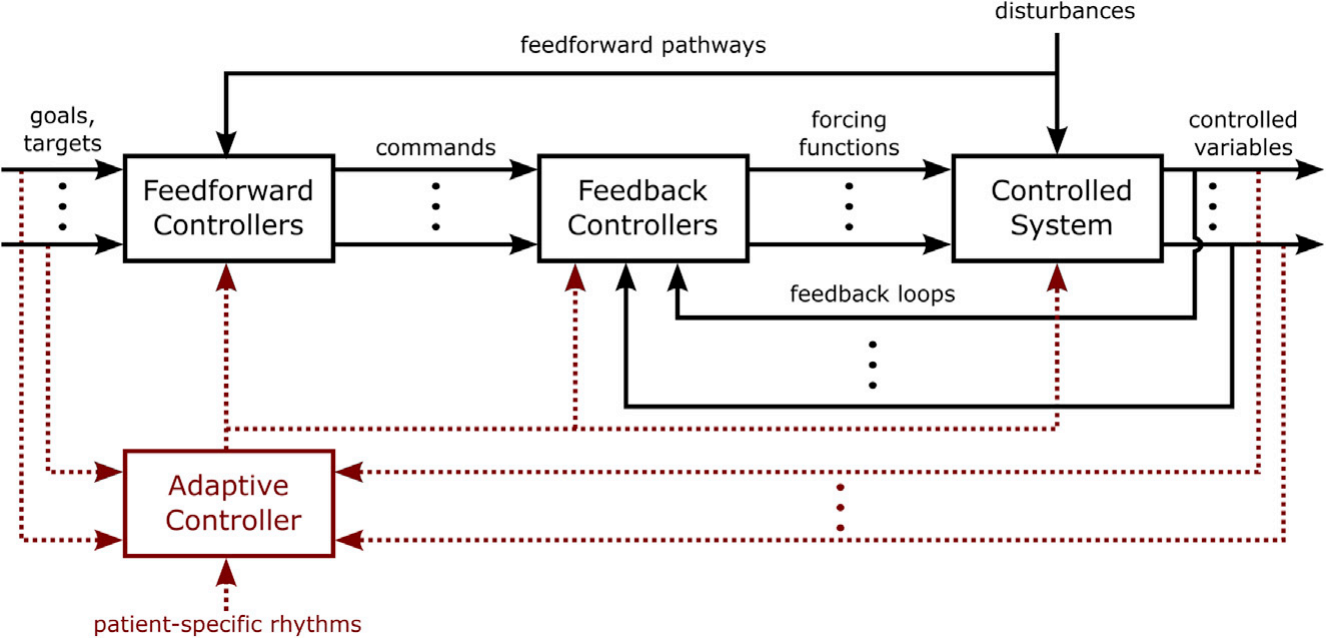
Control Structure for a physiological system Homeostasis in physiological systems is maintained by a combination of adaptive, feedforward, and feedback control strategies. Feedforward and feedback controllers (represented in black) provide time-localized regulation of physiological processes. To maintain homeostasis for time-varying physiological systems, the adaptive controller (represented in red) adjusts elements of the feedforward and feedback controllers in a time-dependent manner in such a way that it is synchronized to patient-specific biological rhythms or in response to slow or intermittent feedbacks. Feedforward and feedback controllers may be implemented using approaches from either classical control theory, such as on-off and proportional-integral-derivative control, or more modern control algorithms, such as fuzzy and model predictive controls. To enable long-term enhancement of future neuromodulation therapies, machine learning and optimization techniques may additionally be incorporated in the adaptive controller to enhance therapy optimization overtime. This figure has been adapted with permission from (Houk, 1988).

In contrast to the definition of adaptive control above, the term ‘adaptive’ is used throughout bioelectronic medicine to describe neuromodulation devices capable of instantaneous parameter adjustments in response to monitored physiomarker signals. These ‘adaptive’ devices in truth rely solely on static implementations of either feedforward, such as activity-based cardiac pacemakers (Dulk et al., 1988; Lloyd et al., 2017) and motion-adaptive spinal cord stimulation devices (Wagner et al., 2018), or feedback control strategies, such as responsive epilepsy stimulation (Geller et al., 2017; Sun and Morrell, 2014) and closed loop deep brain stimulation (DBS) devices (Little et al., 2016; Velisar et al., 2019). These devices do not adjust elements in their control structure to account for longer temporal variations encountered in physiology that may influence therapy outcomes. With improved sensing capabilities and access to longitudinal data in new neuromodulation devices, it is becoming increasingly clear that there is a necessity to realign our definition of ‘adaptive’ devices with those of physiological control systems to optimally regulate disease pathophysiology.

## Biological rhythms and neuromodulation

Although living organisms maintain constant homeostasis, their underlying physiological processes are not time invariant. Physiological processes display rhythmic variations which recur periodically at ultradian (less than 24 h), circadian (24 h), and infradian (>24 h, e.g., 7 days [circaseptan] rhythm time scales) and infradian (1 month) time scales. Circadian rhythms are generated endogenously with a spontaneous period close to but not exactly 24 h. They are synchronized to zeitgebers, or cues from the external environment, the most notable of which is the light-dark cycle (Duffy and Wright Jr, 2005). The light-dark cycle, via the retino-hypothalamic tract, synchronizes the master clock located in the suprachiasmatic nucleus of the ventral hypothalamus, to a 24-h period (Cederroth et al., 2019; Hastings et al., 2019; Kim et al., 2019). This master clock subsequently entrains circadian rhythms in a wide variety of behavioral and physiological variables, such as the sleep-wake cycle, core body temperature rhythm, the cortisol rhythm, cortical excitability, etc. In the absence of the light-dark cycle, and other zeitgebers, the master clock fails to entrain to a 24-h period and results in free-running circadian rhythms, where the endogenous period of the circadian rhythm is greater than 24 h. In rodent animal studies, direct and indirect electrical stimulation of the suprachiasmatic nucleus has been reported to result in phase shifts and period changes in these free-running rhythms (Rusak et al., 1989; Schal et al., 1982). When entrained to a 24-h period, variations in the timing of the sleep-wake and other circadian rhythms are observed between individuals. These variations are referred to as an individual's chronotype, where a chronotype is defined as an individual's natural inclination to sleep at a particular phase of the 24-h day-night cycle (Roenneberg, 2012). Disruption of this sleep-wake circadian rhythm, and more specifically sleep architecture, has been associated with a range of disease pathophysiology (Steele et al., 2021). However, other rhythmic variations are also reported throughout disease pathophysiology, such as endogenous circadian fluctuations in symptom severity (Smolensky et al., 2015) and exogenous ultradian variations induced because of medication scheduling (Smolensky et al., 2021). Due to this, scheduling medication intake based on the time-of-day is an important consideration when optimizing patient-specific therapeutic outcomes, where medication dosages are scheduled to maximize therapeutic benefits and avoid disruption of the circadian sleep-wake cycle (Cardinali and Pandi-Perumal, 2006; Ruben et al., 2019; Smolensky et al., 2021; Smolensky and Peppas, 2007; Videnovic et al., 2014; Zaki et al., 2019). This is further emphasized in medical technologies like the artificial pancreas which provides automatic regulation of blood glucose for patients with Type 1 diabetes (Khodaei et al., 2020). To prevent nocturnal hypoglycemia which may occur during sleep when patients cannot monitor their blood glucose, periodic time-dependent adjustments can be incorporated in the device control strategy to provide safe blood glucose levels over the entire day-night cycle (Gondhalekar et al., 2013).

Due to limited sensing capabilities of previous generations of bioelectronic devices, the influence of biological rhythms on neuromodulation efficacy has been largely underexplored. Traditionally, therapy optimization for bioelectronic devices has been limited to daytime scheduled clinical visits, a single phase of the day-night cycle. As a result, tonic stimulation parameters or control goals for ‘adaptive’ bioelectronic devices may provide effective therapeutic benefits for the specific phase or the day-night cycle that they are optimized for, but may perform better or worse, and in worst cases induce side-effects during other phases of the circadian cycle (Amara et al., 2012; Sharma et al., 2018; Voges et al., 2015). For this reason, time-of-day adjustments to stimulation parameters have been incorporated in some vagal nerve stimulator devices, such as the SenTiva from LivaNova, to minimize therapy side-effects at nighttime. However, this feature is yet to become standard in other bioelectronic devices. In the following sections, we highlight the presence of biological rhythms in preliminary longitudinal data recorded from patients with PD and epilepsy during DBS therapy. Furthermore, we highlight how time-dependent stimulation parameter adjustments synchronized to these rhythms can be implemented to improve patient therapeutic outcomes.

### Case study 1 – Parkinson's disease

Parkinson's disease (PD) is one of the fastest growing neurological conditions with enormous human and economic costs. It is estimated that PD will affect 1.5 million people in the United States and would cost more than $79 billion by 2037 (Yang et al., 2020). Medications are standard therapy for patients, where medication is generally provided in tablet form on a fixed schedule to replace or boost endogenous dopamine (Armstrong and Okun, 2020). However, despite optimal medical care, the majority of patients will experience fluctuations in their motor symptoms over time (Schrag and Quinn, 2000). For these patients, advanced treatment options include dopaminergic infusions or DBS, which can reduce but usually do not eliminate fluctuations (Worth, 2013).

Over time, the management of PD becomes significantly oriented around the optimization of therapy toward symptom fluctuations that occur according to biological and exogenously driven rhythms. Although PD symptoms are described to be improved on waking — the “sleep benefit” (Merello et al., 1997) — overall sleep quality and the sleep-wake circadian cycle in PD is notably disrupted (Mantovani et al., 2018). This is characterized by frequent awakenings and decreased time in both deep (N3) sleep and rapid eye movement stages (REM) as measured by extracranial and intracranial recordings of neural rhythms (Zahed et al., 2021). Treatment for PD is currently optimized for daytime motor control with both medications and DBS titrated to the awake state. Therefore, these treatments also impose intervention-related ultradian rhythms on patients with PD. Dopaminergic medication is generally taken during the day on a fixed schedule between two and four hourly, which results in transitioning from OFF → ON → OFF states throughout the medication cycle. Before bedtime, patients often take a single long-acting dopaminergic medication to treat overnight akinesia, imposing a slow medication cycle overnight, although this has not been found to impact sleep microstructure (Wailke et al., 2011).

Overall, it has been found that open-loop DBS improves sleep structure as a fortuitous byproduct (Hjort et al., 2004; Mizrahi-Kliger et al., 2020). However, it is not currently optimized for nighttime or within sleep stages (Baumgartner et al., 2021; Zahed et al., 2021). Recently, investigational trials have used newer sensing-enabled DBS devices to deliver closed loop DBS in response to biomarkers correlated with motor performance (Gilron et al., 2021; Little et al., 2016; Velisar et al., 2019). These closed loop approaches are currently being oriented around daytime motor fluctuations but may lead to suboptimal therapy and sleep disruption at nighttime if the biomarker used to adjust stimulation is naturally reduced relative to its daytime counterpart (Urrestarazu et al., 2009), Figure 2. We hypothesize that these circadian and rhythmic symptom fluctuations in PD may be better regulated by using a physiological control structure as detailed in Figure 1. An adaptive controller synchronized to patient-specific biological rhythms that are slower and relatively predictable in PD, such as the sleep-wake circadian and ultradian medication cycles, could adjust open-loop stimulation parameters and elements of the feedforward and feedback controllers to improve patient-therapeutic outcomes for each phase of the 24-h day-night cycle (Gilron et al., 2021). In this manner, variations because of sleep-wake and medication cycles could be regulated by synchronized adjustments in patient-specific open-loop DBS parameters. Nonrhythmic, unpredictable, and/or fast disturbances encountered may subsequently be accommodated by feedforward and feedback controllers whose objectives are time-localized to maintain homeostasis at the associated phase of the sleep-wake cycle.

**Figure 2:**
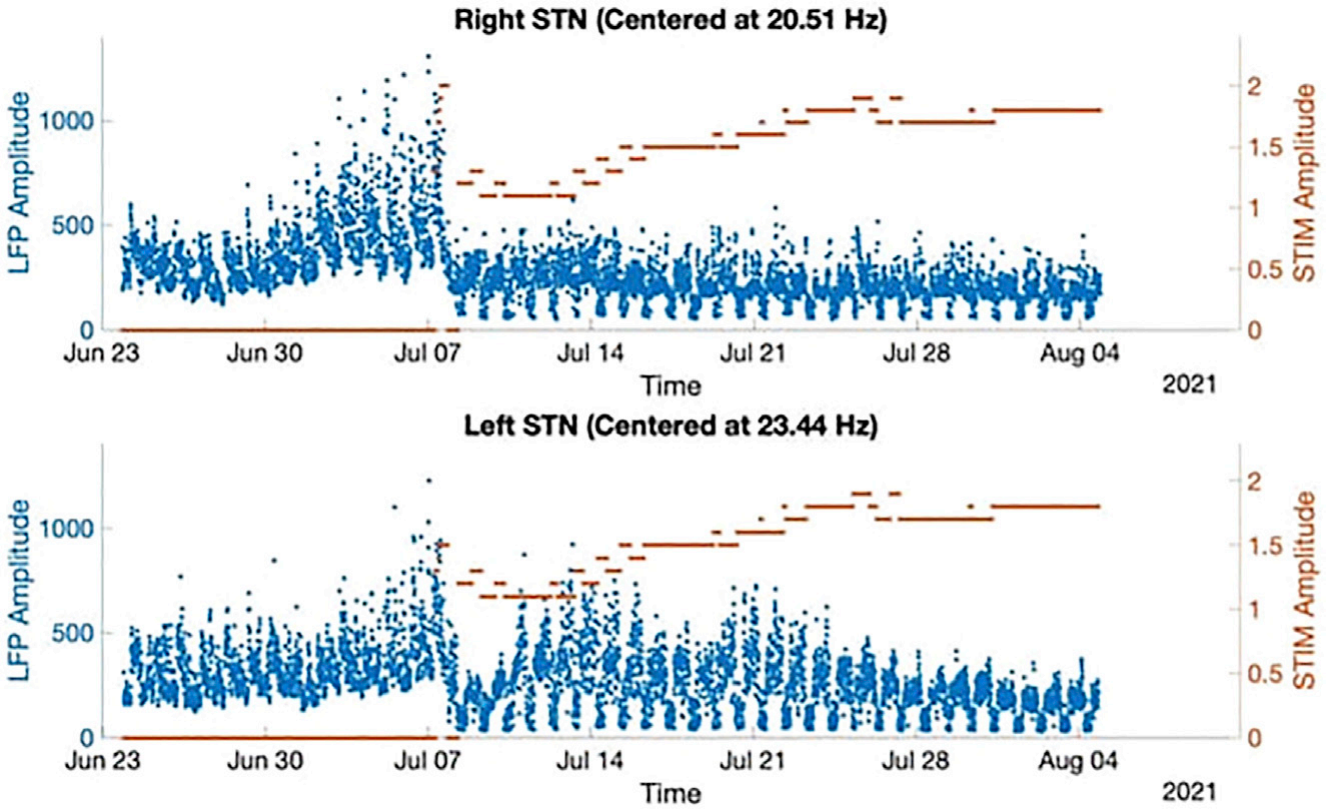
Chronic subcortical recordings from the subthalamic nucleus in a patient with Parkinson's disease Bilateral subcortical recordings from a PD patient implanted with a sensing enabled DBS device, the Medtronic Percept PC™, tracking subcortical beta band power. Note the strong rhythmic circadian fluctuations in beta amplitude over the 24-h cycle, in addition to the influence of stimulation intervention which suppresses beta and compresses the circadian beta fluctuation cycle.

### Case study 2 – epilepsy

Epilepsy is a neurological disease in which brain activity becomes abnormal, resulting in seizures or periods of unusual behavior, sensations, and sometimes loss of awareness. Epilepsy affects close to 50 million people worldwide and has been estimated to cost the United States economy $9.6 billion annually (Yoon et al., 2009). Although antiepileptic drugs are standard medical therapy for patients, around one-third of the patients are refractory to medication and continue to have sporadic seizures. For drug resistant patients, surgical interventions such as resective surgery or DBS are alternative options for seizure management. However, in practice, resective surgery is not suitable for many drug resistant patients because of the epileptogenic brain region generating seizures being poorly localized, originating from multiple foci, or involving brain regions that cannot be safely resected. Furthermore, resective surgery is irreversible and only achieves seizure freedom in approximately 50% of the operated patients overall (Jehi et al., 2015; Téllez-Zenteno et al., 2005). Therefore, DBS is an enticing option for these patients because of its reversible nature, where electrodes can be explanted with minimal side effects if required. Duty cycle stimulation of the anterior nucleus of thalamus (ANT) and responsive neural stimulation, where stimulation is triggered by using detected focal epileptiform activity, have received FDA approval for epilepsy (Bergey et al., 2015; Fisher et al., 2010; Morrell, 2011; Salanova et al., 2015).

However, the optimization of stimulation is difficult and long-term seizure freedom is rare (Bergey et al., 2015; Nair et al., 2020; Salanova et al., 2021). The relevance of circadian rhythmicity in epilepsy has been recognized (Khan et al., 2018) but because of technological limitations of neuromodulation devices to-date, few clinical studies have investigated the rhythmicity and periodicity of seizures and their associated epileptiform brain activity. Therefore, optimization of patient-specific stimulation parameters has been primarily driven by treatment failures estimated from seizure diaries collected by patients and their caregivers. However, the use of these diaries for monitoring seizure outcomes has been shown to demonstrate great inaccuracy. In combination with the infrequent nature of seizures, where patients spend most of their time in a non-seizure state, physicians are thus required to optimize therapy over extended periods of time (Bergey et al., 2015; Blachut et al., 2015, 2017; Hoppe et al., 2007; Salanova et al., 2015). Access to newer sensing-enabled devices capable of chronic data recording are highlighting a clearer role of biological rhythms in epilepsy (Baud et al., 2018; Gregg et al., 2020, 2021; Karoly et al., 2021). More specifically, long-term intracranial EEG (iEEG) recordings from human and canine patients demonstrate electrophysiological biomarkers with circadian and infradian seizure periodicities that occur independent of medication dosing that are believed to reflect endogenous rhythms associated with seizure risk (Baud et al., 2018; Gregg et al., 2020; Karoly et al., 2018), Figure 3.

**Figure 3:**
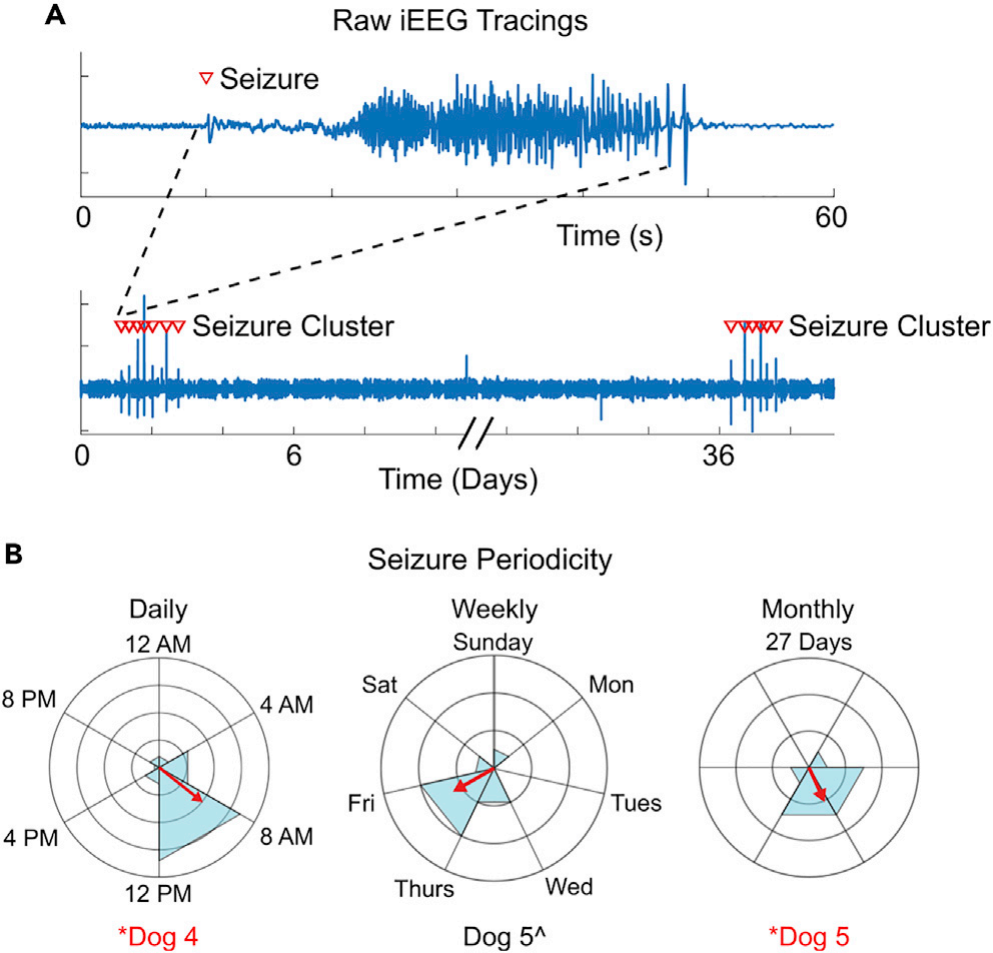
Example seizure periodicity in canine epilepsy (A) Raw iEEG tracings. iEEG tracings are displayed at multiple timescales to illustrate a single seizure and a pair of seizure clusters separated by several days. Red triangles indicate seizure onset. (B) Circadian, circaseptan, and monthly seizure periodicity. Daily, weekly, and monthly circular histograms of seizure occurrence. Concentric rings demarcate the number of seizures (five seizures per concentric ring in the daily histogram, two seizures per ring in the weekly and monthly histograms. The red bar is the resultant vector or R value. Dog four and Dog five showed statistically significant daily and monthly periodicity, respectively, as indicated by the red font and asterisk. Dog five also indicates a trend toward significant weekly periodicity;’ however, this did not survive statistical false discovery rate correction. This figure by (Gregg et al., 2020) is licensed under CC BY-NC 4.0 and adapts Figures 1B and 3 from their original publication.

Disruption of the sleep-wake cycle is also common in epilepsy; however, stimulation at present is not optimized for nighttime or within sleep stage operation (Moore et al., 2021; Ruoff et al., 2020; Voges et al., 2015). ANT stimulation has been reported to disrupt sleep in a voltage-dependent manner, where the frequency of arousals during sleep is positively correlated with the DBS voltage (Voges et al., 2015). Nighttime modulation of the DBS amplitude has thus been suggested as an approach to mitigate sleep architecture disruption during ANT stimulation. Similarly, patients with vagal nerve stimulators have been reported to demonstrate increased incidences of sleep apnea (Parhizgar et al., 2011). Due to this, vagal nerve stimulator devices such as the SenTiva from LivaNova have incorporated time-of-day based stimulation parameter adjustments to overcome this side effect. Future stimulation devices for epilepsy may further improve therapeutic outcomes if stimulation parameters and control goals are dynamically adapted in this manner to reinforce sleep and other healthy brain rhythms. These devices may improve regulation of physiological brain activity to minimize seizure frequency for patients.

## Future adaptive bioelectronic devices

The above case studies illustrate two examples of neurological disorders which demonstrate strong rhythmic variations across several timescales. Many other biological processes and neurological disorders are reported to display similar variations. For example, brain temperature, cortical excitability, and brain responses to a performance task are all modulated by circadian and sleep-wake cycles (Landolt et al., 1995; Ly et al., 2016; Muto et al., 2016); the circadian sleep-wake cycle modulates the threshold for pain perception (Hagenauer et al., 2017), while a variety of headache disorders are also synchronized to certain phases of the sleep-wake cycle (Burish et al., 2019; Naber et al., 2019). Patients with schizophrenia demonstrate significant disruption of circadian rhythms and sleep (Wulff et al., 2012), and there is considerable evidence of altered circadian rhythms, sleep disturbances, and diurnal mood variation in patients with depression (Germain and Kupfer, 2008; Logan and McClung, 2018; Monteleone et al., 2011). Taken together, these studies suggest that future bioelectronic devices may improve patient therapeutic outcomes by integrating biological rhythm considerations into their control structures and realigning their implementations of adaptive control with the physiological control structure presented in Figure 1. More specifically, we propose that future neuromodulation devices should incorporate feedforward, feedback, and adaptive controllers to regulate disease pathophysiology, where the implemented adaptive controller is synchronized to patient-specific rhythms, such as the patient's chronotype and medication schedule. In this manner the role of the adaptive controller is to adjust the device therapeutic control objectives based on the instantaneous phase of these rhythms, whereas the feedforward and feedback controllers regulate disease pathophysiology and intervention related side effects in a time-localized manner by providing anticipatory and instantaneous adjustments to stimulation parameters. Overall integration of this physiological control structure in future devices would enable optimization of therapeutic outcomes over both short and long-time scales. To illustrate this chronoadaptive methodology, in the following sections we highlight examples of initial implementations of chronotherapeutic DBS devices taken from our recently published work.

### Preliminary implementations of chronoadaptive bioelectronic devices

We have undertaken preliminary work to integrate patient-specific rhythms in DBS therapy. In the following sections, we briefly highlight this work in two investigational DBS devices for the treatment of PD and epilepsy.

#### Investigational medtronic summit RC + S™ – sleep-aware adaptive DBS for PD

**Figure 4:**
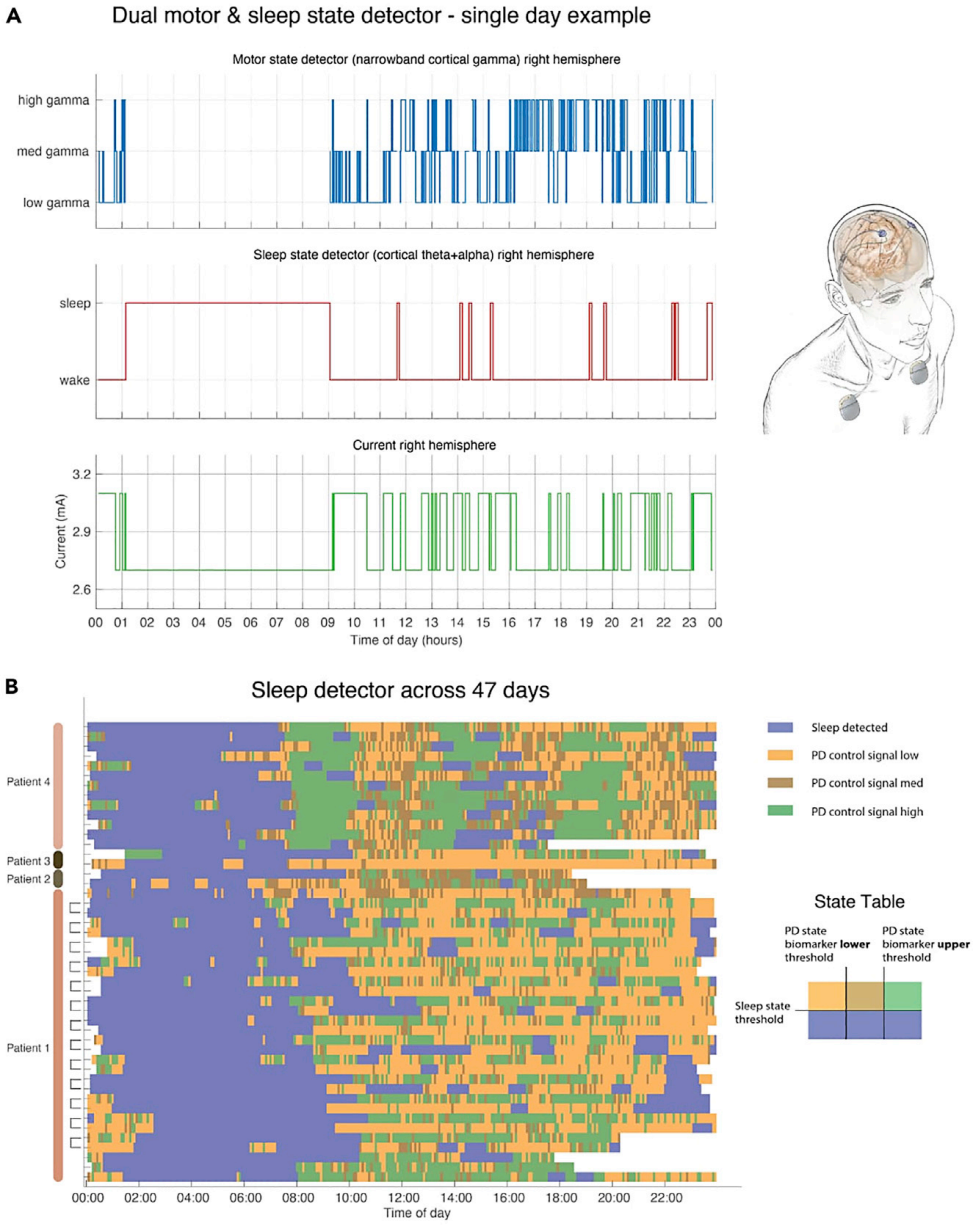
Fully-embedded sleep adaptive closed loop DBS control in a patient with PD (A) 24 h performance of dual motor and sleep state classifiers for closed loop amplitude modulation. When the sleep classifier detects sleep, the motor state classifier is disabled and fixed amplitude ope loop stimulation is applied. Otherwise, when sleep is not detected, the motor state classifier increases or reduces the stimulation amplitude when the monitored cortical gamma activity is low or high, respectively. (B) Sleep detector performance over 47 days. The heatmap summarizes the sleep and motor state classifier performance over 47 days measured across four patients. Blue boxes indicate periods classified as sleep, whereas yellow and green boxes indicate the motor state as summarized in the state table. This figure by Gilron et al., 2021 is licensed under CC BY 4.0 and combines Figures 2A and 4A from their original publication.

In Gilron et al., 2021, we presented an embedded sleep-aware adaptive DBS control system for PD implemented on an investigational Medtronic RC + S implantable pulse generator (IPG). In this study, four patients with PD were implanted bilaterally with cylindrical DBS leads in the subthalamic nucleus and paddle-type quadripolar leads in the subdural space over the motor cortex. The cortical and subcortical leads from each side were connected to an IPG which allowed independent control of each hemisphere. Two embedded classifiers on the IPG were programmed for the dual detection of neurophysiological biomarkers associated with sleep and the parkinsonian motor state, respectively, Figure 4A. The motor state classifier estimated “on” and “off” medication states for patients, where stimulation was reduced during on states to avoid stimulation induced dyskinesia and increased during off states to improve patient mobility (Gilron et al., 2021). When sleep was detected by the sleep classifier, the IPG delivered constant stimulation equivalent to clinically optimized open-loop stimulation regardless of the state of the motor state classifier. The interaction between the two classifiers was captured as a state table on the IPG, Figure 4B. The performance of the classifiers was verified using patient motor diaries, wearables, and the RC+S onboard accelerometer. In cases where the dual classifiers were deployed, the mean concordance between sleep measurements across both hemispheres was 88% (range 77–98%). In addition, we reported stable performance of the dual classifiers when tested over the course of 47 patient-days (24 h) across four patients and six hemispheres. To assess the utility of a dedicated sleep classifier, we also simulated algorithm performance with omission of the sleep classifier. With its omission, all patients displayed large variation in the stimulation control signal during periods of sleep which could result in unwanted behavior depending on the algorithm used to control PD motor states during waking hours.

#### Investigational medtronic summit RC + S™ - day/night DBS scheduling for epilepsy

**Figure 5:**
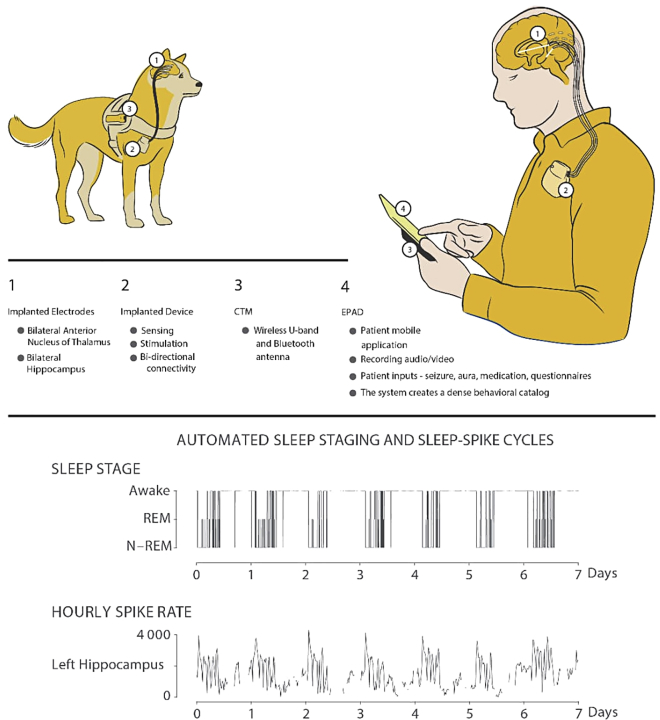
The Mayo EPAD distributed brain coprocessor system for daytime/nighttime algorithm scheduling The EPAD system provides bidirectional communication between implantable neuromodulation devices and commercially available electronics. The system has been investigated in both human and canine patients with epilepsy. The system is capable of sleep stage detection based on recorded neurophysiological data and enables targeted adaptation of stimulation during sleep.

Sleep comorbidities are commonly experienced by patients with epilepsy. To improve sleep quality and seizure mitigation for patients with epilepsy, we presented the Mayo Epilepsy Personal Assistant Device (EPAD) in (Attia et al., 2021; Sladky et al., 2021). The EPAD system is a distributed brain coprocessor system that integrates implantable brain sensing and stimulation devices with off-the-body commercial electronics for clinical and neuroscience research applications (Attia et al., 2021; Sladky et al., 2021; Figure 5). The system overcomes computational and data storage limitations of bioelectronic devices with fully-embedded algorithms by providing wireless bidirectional connectivity between local tablet processors and distributed cloud computing technology (Kremen et al., 2018). We investigated the distributed system for the treatment of drug-resistant epilepsy in both canine and human patients (Attia et al., 2021). The distributed system enabled continuous electrophysiology data streaming to a local tablet computer for real-time analysis and tracking of interictal epileptiform spike, seizures, and brain behavioral state coupled with patient reports to inform automatic adjustments of DBS parameters to optimize patient therapeutic outcomes (Attia et al., 2021; Kremen et al., 2018; Mivalt et al., 2021; Stanslaski et al., 2018). In prospective data recorded from one human and two canine ambulatory subjects, the interictal epileptiform spike detection algorithm, when compared to gold standard expert visually reviewed events, resulted in a sensitivity value of 0.9 and an F1-score of 0.81 (Sladky et al., 2021). We also reported impressive performance from the device seizure detector with area under the curve, precision, and recall values of 0.93, 0.47, and 0.88 for the human and two canine subjects, respectively (Sladky et al., 2021). The distributed system enabled the deployment of circadian adaptive control strategies where daytime stimulation parameters (amplitude and frequency) were adjusted to nighttime parameters optimized for minimizing sleep disruption (Attia et al., 2021; Sladky et al., 2021).

#### Picostim DyNeuMo-1 - day/night-DBS control scheduling for epilepsy

**Figure 6:**
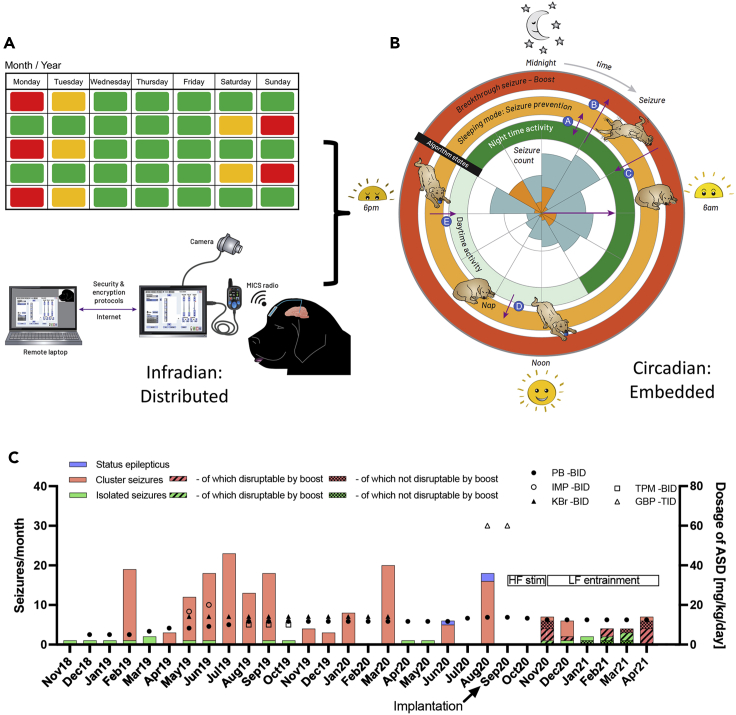
Example of DBS chronotherapy for canine epilepsy (A) Infradian stimulation scheduling. The Picostim DyNeuMo-1 system enables time-based scheduling of stimulation adjustments synchronized to the patient's particular infradian seizure rhythm (e.g., a 2 weekperiod). (B) Rose plot illustration of embedded circadian stimulation algorithm adjustments. The inner circle represents the seizure count from the patient diary; the orange tiling is the timing of first seizure onset, whereas the blue accounts for all seizures in a cluster. The algorithm is composed of three states (represented as rings) to facilitate day/nighttime-based stimulation scheduling (inner green ring), a motion-triggered sleep mode to prevent seizure occurrence during daytime napping (middle pale orange ring), and a tap-activated boost mode for preventing breakthrough seizures (outer dark orange ring). (C) Summary of canine seizure frequency and anti-seizure drug dosage preimplantation and postimplantation. Postimplantation, the canine experiences no status epilepticus events and no significant seizure clusters thus resulting in a reduction in rescue medication. This figure by (Zamora et al., 2021) is licensed under CC-BY 4.0 and combines Figures 3 and 4A from their original publication.

In Zamora et al., 2021, we investigated circadian adaptive DBS therapy in a case study of a canine with severe drug-resistant idiopathic generalized epilepsy that exhibited a characteristic nocturnal pattern correlated with its sleep-wake cycle. Before DBS device implantation, the canine's cluster seizures evolved to status epilepticus and required emergency pharmacological intervention. The canine was implanted with a cranial-mounted DBS system (Toth et al., 2020; Zamora et al., 2020), whose electrodes were implanted bilaterally in the centromedian nucleus of the thalamus. The DBS system implanted in this case study was capable of both motion and time-based stimulation parameter adjustments fully-embedded on the device, as detailed in Toth et al., 2020; Zamora et al., 2020 and illustrated below in Figure 6B. The DBS device was configured to deliver stimulation at specific frequencies characteristic of healthy neurophysiological activity and provide time-based stimulation amplitude adjustments synchronized to the patient's specific nocturnal and infradian (2-week period) seizure rhythms. Time-based adjustments were supplemented by stimulation adjustments in response to activity to mitigate seizures during daytime napping and to interrupt potential breakthrough seizures. Post implantation, administration of the rescue medication levetiracetam was initially continued as a pulse therapy after seizure occurrence to prevent cluster seizure evolution or the occurrence of status epilepticus. In seven periods, stimulation without rescue medication was successful in disrupting cluster seizure emergence. The medication, Phenobarbital, was continued as chronic treatment over the whole observation time after implantation with a dose reduction from 13.3 to 12.5 mg/kg/day in November 2020. At the time of writing this case study (7 months), the canine had experienced no further status epileptic events and no significant seizure clusters, Figure 6C. Further details regarding medication and stimulation parameters implemented during the case study can be found in the original publication (Zamora et al., 2021).

## Future states and limitations

The above examples highlight potential technological infrastructures required for integrating chronotherapy in bioelectronic devices. In practice, future devices will require hybrid frameworks to leverage the benefits of both embedded and distributed device functionalities. In this manner flexible device platforms will provide clinicians with improved insight into disease pathophysiology across a breadth of neurological disorders, while facilitating long-term optimization of neuromodulation chronotherapy on a disease-specific and patient-specific basis. Furthermore, flexible platforms such as this would facilitate exploration of alternative algorithms for regulating stimulation parameters in accordance with the relevant biological rhythms. On-off and proportional-integral-derivative strategies from classical control theory may be readily suitable for embedded implementations on current device hardware. In contrast, implementations of modern control or machine learning algorithms which are computationally expensive, such as Bayesian optimization and reinforcement learning (Grado et al., 2018; Lu et al., 2020), and may require distributed infrastructures because of the power and data storage limitations of current device technology. In addition, incorporating distributed functionalities in future bioelectronic devices will enable the tracking of algorithm’s therapeutic performance over time, to be reviewed by clinicians at scheduled patient visits, and allow intermittent, wireless algorithm updates to be deployed over the cloud as necessary to provide seamless improvements in therapy performance (Kremen et al., 2018).

However, limitations may also be encountered in the development of these future chronotherapeutic-bioelectronic devices. The ability to trial new computationally expensive algorithms comes with an increased algorithm training complexity. Moreover, the ability to adapt the control strategy or stimulation parameters implemented in response to specific phases of biological rhythms may also result in increased clinical burden and programming time requirements. However, as highlighted in this article, the state-of-the-art for many neuromodulation therapies at present is still oriented around the delivery of open-loop stimulation optimized for maximizing daytime therapeutic benefits. Therefore, we propose that first generation chronoadaptive-bioelectronic devices may readily improve patient therapeutic outcomes by simply implementing time-based adjustments to open-loop stimulation parameters aligned to patient-specific chronotypes and medication schedules. Following this, if further therapy refinement is necessary, additional feedforward-based or feedback-based strategies whose control goals are appropriately synchronized to specific phases of these patient rhythms can be gradually layered in to provide additional stimulation refinement in a time-localized manner, only when required. This framework should help to constrain the multiple degrees of freedom encountered when developing new strategies for automatic therapy optimization in a tractable manner. At a minimum, deployment of chronoadaptation in this manner should immediately enhance the therapeutic benefits of open-loop stimulation at nighttime during sleep, while maintaining the performance of daytime optimized therapy. Trialing of future feedforward-based and feedback-based control strategies may then focus on optimization of therapy goals during specific phases of the day-night cycle.

## Conclusions

With the continued development of bioelectronic device technology, previous technical limitations that have limited access to longitudinal data recordings from patients with neurological disorders in the past are gradually being eased. As a result, clinical neuroscience researchers are beginning to gain insight into rhythmic variations in patient physiology over a variety of timescales and explore the influence of these biological rhythms on the therapeutic outcomes of neuromodulation therapies. With this in mind, it is becoming increasingly clear that there is a need to incorporate considerations for these biological rhythms in future bioelectronic devices. To optimize patient-specific therapeutic outcomes, we emphasize that future devices should incorporate feedforward, feedback, and adaptive control strategies together to maximize therapeutic benefits for patients and provide more natural regulation of patient pathophysiology to restore healthy physiological homeostasis.
